# *In vivo* microstructural investigation of the human tympanic membrane by endoscopic polarization-sensitive optical coherence tomography

**DOI:** 10.1117/1.JBO.28.12.121203

**Published:** 2023-03-29

**Authors:** Svea Steuer, Joseph Morgenstern, Lars Kirsten, Matthias Bornitz, Marcus Neudert, Edmund Koch, Jonas Golde

**Affiliations:** a TU Dresden, Department of Medical Physics and Biomedical Engineering, Faculty of Medicine, Dresden, Germany; b TU Dresden, Anesthesiology and Intensive Care Medicine, Clinical Sensoring and Monitoring, Faculty of Medicine, Dresden, Germany; c TU Dresden, Otorhinolaryngology, Ear Research Center Dresden, Faculty of Medicine, Dresden, Germany; d TU Dresden, Else Kröner-Fresenius Center for Digital Health, Dresden, Germany

**Keywords:** optical coherence tomography, polarimetry, eardrum, birefringence, *in vivo*, endoscope

## Abstract

**Significance:**

Endoscopic optical coherence tomography (OCT) is of growing interest for *in vivo* diagnostics of the tympanic membrane (TM) and the middle ear but generally lacks a tissue-specific contrast.

**Aim:**

To assess the collagen fiber layer within the *in vivo* TM, an endoscopic imaging method utilizing the polarization changes induced by the birefringent connective tissue was developed.

**Approach:**

An endoscopic swept-source OCT setup was redesigned and extended by a polarization-diverse balanced detection unit. Polarization-sensitive OCT (PS-OCT) data were visualized by a differential Stokes-based processing and the derived local retardation. The left and right ears of a healthy volunteer were examined.

**Results:**

Distinct retardation signals in the annulus region of the TM and near the umbo revealed the layered structure of the TM. Due to the TM’s conical shape and orientation in the ear canal, high incident angles onto the TM’s surface, and low thicknesses compared to the axial resolution limit of the system, other regions of the TM were more difficult to evaluate.

**Conclusions:**

The use of endoscopic PS-OCT is feasible to differentiate birefringent and nonbirefringent tissue of the human TM *in vivo*. Further investigations on healthy as well as pathologically altered TMs are required to validate the diagnostic potential of this technique.

## 1 Introduction

Optical coherence tomography (OCT) is an interferometric imaging technique based on near-infrared light that enables real-time volumetric tissue imaging with microscopic resolution and penetration depths of up to 2 mm. While OCT has been successfully commercialized in ophthalmology for diagnostics of the eye, the application of both morphologic and functional OCT imaging for hearing diagnostics has attracted significant interest in recent years.^1^ This includes not only the tympanic membrane (TM)^2^^,^^3^ but also the ossicular chain in the middle ear^4^^–^^6^ and the inner ear.^7^^,^^8^

The eardrum or TM of conical shape separates the ear canal from the middle ear and receives incoming sound waves. It is connected to the malleus of the ossicular chain, whereby vibrations of the TM are conducted to the inner ear mechanically by the following ossicles incus and stapes. The main region of the membrane is the pars tensa surrounded by the annulus, a fibrocartilaginous ring, whereas the minor part, the pars flaccida, with only loose connective tissue is found superior to the malleus handle. The structure of the pars tensa is described to be composed of three layers:^9^ an epidermal layer at the lateral side, followed by the lamina propria, and a thin mucosal layer at the medial side. Light microscopic and electron microscopic surveys^9^^–^^11^ have shown that the lamina propria consists of a subepidermal and a submucosal connective tissue layer embedding outer radial and inner circular collagen fibers. Funnel and Laszlo^12^ summarized that the radial fibers have a higher density converging to the malleus, and that the circular fibers are less or not apparent in the central region of the TM.

While only limited insights onto the thickness of the lamina propria, especially in humans, are reported, the full thickness distribution of the human TM was evaluated from *ex vivo* OCT measurements by Van der Jeught et al.^13^ Thickness variations are widely spread across individual TMs. However, the trend of thinnest central regions in the order of 50 to 70  μm and a thickness increase toward the annulus ring and toward the connection to the malleus of up to 100 to 120  μm is generally confirmed in all samples. *In vivo* thicknesses observed with endoscopic OCT were provided recently^14^ as an average over selected points within the central area of the TM and a range of 112 to 127  μm.

In general, the acoustical and mechanical properties of the TM are a critical part of the auditory system and depend largely on the scaffold of the lamina propria.^15^^,^^16^ The importance of the collagen layer for sound conduction was shown by experiments on intact TMs of temporal bones.^17^ There, the TM function was modified by cutting slits into the TM, which was subsequently covered with paper patches. Hence, a reduced ability of conducting frequencies above 4 kHz was observed, which indicates how the frequency-dependent hearing ability is altered when collagen structures of the TM are affected. Complementary, pathological TMs exhibit changes of the regular arrangement of collagen fibers as, for example, the formation of fiber bundles was observed by Hiraide et al.^18^ and related to the healing process following an inflammation.

Besides this broad foundation from anatomical and physiological studies, Mozaffari et al.^19^ stated a need in directly applicable imaging technology for a better understanding of TM-related pathogenesis and TM development in general. In addition, latest research in the field of three-dimensional (3D) bioprinting addresses a demand by Funnel and Laszlo^12^ from the 1980s, postulating the “evaluation of methods of corrective middle-ear surgery, especially the design of drum protheses.” Recently, von Witzleben et al.^20^ demonstrated a biomimetic TM replacement based on melt electrowriting but with the restriction to linearly aligned fibers for practical reasons. Trying to reproduce the TM fiber structure and properties more precisely, Anand et al.^21^ noticed that questions regarding the “independent contribution of radial versus circumferential collagen fibers” still remain largely unanswered. Accordingly, ongoing preclinical research raises the need for imaging technologies that facilitate the assessment of the TM and its microstructure.

Different optical technologies provide a connective tissue-specific contrast, e.g., due to the anisotropy and birefringence of collagen fibers, which is most frequently exploited by means of polarized light microscopy.^22^ Depending on the resolution, this technique can be used to assess the retardation and optic axis orientation of individual fibers or fiber bundles on a larger scale but is typically limited to projection measurements in transmission. Compared to that, utilizing the second-harmonic contrast in multiphoton microscopy enables 3D imaging of the individual fibers and has been used to study the TM collagen structure^23^^,^^24^ but is, due to the required illumination characteristics in nonlinear microscopy, practically not applicable *in vivo*.

Polarization-sensitive OCT (PS-OCT), an extension of OCT that utilizes the contrast from polarization changes in tissue similarly to polarized light microscopy, has the potential to bridge the gap between those modalities and real-time *in vivo* imaging applications. While PS-OCT has been demonstrated for manifold applications including the retina,^25^ oral soft,^26^ and hard tissue^27^ as well as intravascular imaging^28^^,^^29^ and breast tissue,^30^ first preliminary results on the TM were provided quite recently.^31^^,^^32^ While endoscopic OCT for TM and middle ear diagnostics has been successfully demonstrated using gradient index (GRIN) lenses as endoscope optics,^2^^,^^33^ PS-OCT has so far not been applied with GRIN imaging optics. The manufacturing process of a GRIN lens, typically an ion-exchange process, inserts not only a refractive index profile in a radial direction, which enables imaging, but also induces birefringence.^34^^,^^35^ For imaging through a GRIN lens, a distribution of polarization states over the field of view is implied, even though illuminated with a distinct input polarization state, which can be utilized for certain polarimetric applications^36^ but is commonly unfavorable for PS-OCT imaging and conventional processing.

Here, we describe the modifications of a previously developed endoscopic OCT system to perform PS-OCT measurements of the human TM *in vivo* and a Stokes-based processing approach to determine a local retardation contrast. By examining both ears of a healthy volunteer, we demonstrate the feasibility of the developed endoscopic PS-OCT system in assessing the connective tissue and thus, the layered microstructure of the TM as well as the limitations therein. Finally, we discuss the current limitations, both due to the anatomical constraints and due to the system properties, as well as strategies to address these for potential clinical applications and successful translation.

## 2 Materials and Methods

### 2.1 Endoscopic PS-OCT System Setup

**Fig. 1:**
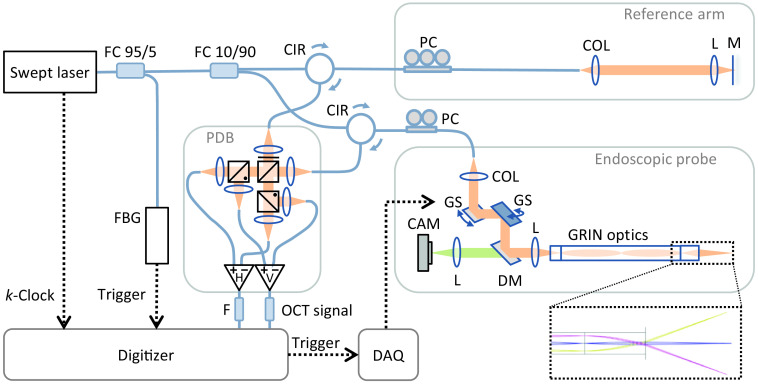
Endoscopic PS-OCT setup with fiber couplers (FC), optical circulators (CIR), fiber collimators (COL), lenses (L), polarization controllers (PC), and a mirror (M) in the reference arm, galvo scanners (GS) as well as a dichroic mirror (DM), and a visible-light camera (CAM) in the sample arm. An FBG module provides a phase-stable A-scan trigger for digitizing the low-pass filtered (F) polarization-diverse balanced (PDB) detection signal.

The endoscopic PS-OCT system used in this investigation is based on the setup described by Kirsten et al.^2^ The revised system was built with a swept-wavelength laser source (SL132120, MEMS-VCSEL, Thorlabs) operating at a sweep rate of 200 kHz, a center wavelength of 1300 nm, and an effective depth range of 8 mm due to the optical delay in the ancillary interferometer, which is used for linear-in-wavenumber sampling, i.e., the k-clock integrated in the source. Ninety-five percent of the emitted light was directed into a Mach–Zehnder interferometer, whereas the residual 5% was used in a fiber-Bragg grating (FBG) module to create an A-scan trigger signal. The interferometer contained a fiber coupler splitting the light in the ratio 10:90. Two optical circulators led the minor part of the light to the reference arm and the major part to the sample arm containing the endoscopic probe. The reference arm consisted of a fiber collimator in front of an achromatic lens and a mirror for retroreflection. In the sample arm, the collimated beam was deflected by two galvanometric scan mirrors to produce cross-sectional and volumetric scans. Simultaneous acquisition of a camera image was realized with visible light by the following dichroic mirror, which reflects the OCT light and transmits the visible part. A Hastings triplet focused the laser beam with a telecentric field onto a GRIN optics persisting of a glass spacer, a GRIN relay lens, and a GRIN objective lens. Thus imaging at the distal end of the endoscopic optics was done with a working distance of 10 mm and a field-of-view (FOV) diameter of 10 mm, which enables examining the whole TM but requires a fan-shaped scanning as visualized by the optical simulation in the dashed box in Fig. 1. This corresponds to an angular FOV of ±30 deg through the GRIN endoscope, which has a diameter of 2 mm. Including the surrounding illuminating fibers for visible-light imaging and a protective metal shell, the outer diameter of the entire endoscopic tube is 3.5 mm.

To adjust the polarization state of the incident and returning light through the single-mode fibers (SMF), polarization-controlling paddles were integrated in both arms. The amount of glass added by the GRIN optics caused a dispersion mismatch, although the chosen fiber length in the reference and sample arm compensated the effect to some extent. The remaining dispersion was software compensated during postprocessing.

The most important modification, compared to the system used beforehand, is the replacement of the detection unit to implement a polarization-sensitive system. Therefore, the back-reflected sample and reference light were guided by fiber collimators to the free-space polarization-diverse balanced detection unit. The polarization of the reference arm was controlled to be diagonally polarized by a polarizer placed at 45 deg in the optical path. In the following, the light beams of the two interferometer arms were superimposed at a 50:50 nonpolarizing beam splitter. The outgoing light was then separated in orthogonally polarized parts by two adjacent polarizing beam splitters. The optical outputs were detected separately for each polarization channel by two identical balanced photoreceivers (PDB470C, Thorlabs). The resulting differential signals were low-pass filtered and acquired by a high-speed digitizer (ATS9373, Alazar Technologies), which used both the provided k-clock of the laser as the sampling trigger and the additional FBG signal for phase-stable A-scan triggering. A custom LabView (National Instruments) software was used for controlling the galvo scanners via analog signals provided by a multi-function digital acquisition board (DAQ), the camera, and OCT preview as well as the data acquisition.

### 2.2 Measurements

The study followed the tenets of the Declaration of Helsinki and was approved by the Institutional Review Board (IRB00001473) at the TU Dresden (EK 252062017). TM examinations utilizing the endoscopic PS-OCT system were conducted by an experienced otolaryngologist in the clinical setting similar to generally performed otoscopy. Volumetric scans were acquired of the left and right ear of a healthy volunteer (female, 29 years). The subject sat on a common examination chair adjustable in height, the examiner on a doctor’s chair with an elbow rest for stabilization. The endoscope was inserted in the ear canal while the positioning was visually controlled by the provided camera image and a preview of orthogonal OCT cross sections. For volumetric scanning, the proximal surface of the GRIN optics was scanned with 500×500 A-scans within < 4  s, accounting for ∼450 A-scans in each lateral direction of the angular FOV of ±30  deg at the distal end. Thereby, a field larger than necessary was acquired to compensate for some flexibility of the endoscopic tube.

After Fourier transform processing, the axial range of 8 mm corresponds to 1024 samples and provides an axial resolution of around 15  μm. Due to the fan-shaped scanning, the lateral sampling is depending on the axial distance but corresponds to a Nyquist sampling of around 22  μm at the working distance of 10 mm for a lateral resolution in focus of ∼45  μm.

**Fig. 2:**
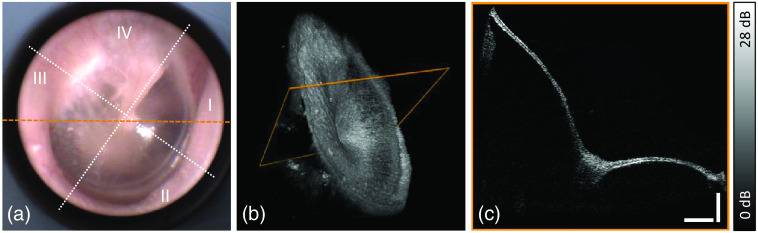
Measurement of the right ear of a healthy volunteer. Visible-light camera image (a) indicating positions of the cross-sectional scans with assignment of the four quadrants of the TM, visualization of the whole volume (b) with animation available online (Video 1), and the cross section (c) corresponding to the marking in (b). For geometrically correct display, a distortion correction algorithm based on Ref. 2 was applied. Scale bars correspond to 1 mm in air (Video 1, MP4, 2.8 MB [URL: https://doi.org/10.1117/1.JBO.28.12.121203.s1]).

Figure 2 illustrates (a) the camera image; (b) the volume of the right TM, which is also provided as an animated version online; and (c) a cross section whose position is marked in orange in Figs. 2(a) and 2(b). In the camera image, the malleus is visible as the border between the posterior superior (IV) and the anterior superior (I) quadrant. The characteristic light reflex as known from standard otoscopy is located close to the center, partly in the anterior superior (I) and partly in the anterior inferior (II) quadrant. The ossicular structures are faintly visible through the translucent membrane, whereas stronger OCT signals in the posterior inferior (III) region were assigned to the promontory wall, separating the middle ear from the inner ear. Structures behind the TM are furthermore expected in the posterior superior quadrant including the incus and the stapes, and in quadrants I and IV the chorda tympani, which is a branch of the facial nerve that runs through the middle ear.

### 2.3 Data Processing

The measured balanced detection signals were processed following the conventional procedure for swept-source OCT data (zero-padding, compensation of the dispersion mismatch, and Hann windowing). The Fourier transform provided complex-valued, depth-resolved A-scans a(z) for both orthogonal polarization directions, here denoted as H and V without loss of generality. Thus the measured signal can be described as a Jones vector:

$${\overset{\to }{e}}_{p}=(\begin{array}{c} a_{H}\\ a_{V} \end{array})=(\begin{array}{c} A_{H}e^{i\varphi_{H}}\\ A_{V}e^{i\varphi_{V}} \end{array}),$$ (1)

where $A_{H,V}$ are the amplitudes and $\varphi_{H,V}$ the phases of each component. Using only the phase difference $\Delta \varphi =\varphi_{V}-\varphi_{H}$ of both components with −π<Δφ≤π, the measured data can be converted to Stokes parameters^37^ by

(S0S1S2S3)=(AH2+AV2AH2−AV22AHAV cos Δφ2AHAV sin Δφ)=(|aH|2+|aV|2|aH|2−|aV|22 Re(aH*aV)2 Im(aH*aV)). (2)

From this, the logarithmic intensity can be directly calculated as

$$I_{\mathrm{dB}}=10\cdot \log_{10}(A_{H}^{2}+A_{V}^{2})=10\cdot \log_{10}(S_{0}).$$ (3)

To evaluate the polarization information, the four Stokes parameters $S_{i}$ were spatially averaged using a 3D Gaussian filter kernel w with pixel size (K,L,M)=(9,13,13) and full-width at half-maximum of (K,L,M)=(4,4,4) so that

$$\overset{¯}{S_{i}}(z,x,y)=\underset{k,l,m}{\sum }w(k,l,m)\cdot S_{i}(z+k,x+l,y+m),$$ (4)

where $k=[-\frac{K-1}{2},\frac{K-1}{2}]\cdot \Delta z$, $l=[-\frac{L-1}{2},\frac{L-1}{2}]\cdot \Delta x$, and $m=[-\frac{M-1}{2},\frac{M-1}{2}]\cdot \Delta y$. As both the axial and the lateral directions were sampled at approximately Nyquist rate, which resulted in a voxel size of Δz=7.8  μm in axial and Δx=Δy=0.13  deg in both lateral directions of the measured data, this averaging kernel included $∼2^{3}=8$ speckles.^38^ This was found to be the lower boundary of a subjectively assessed polarization contrast with the aim of determining a reliable polarization state from the noise and speckle-dependent Stokes parameters derived from the measured data. Then the four Stokes parameters were normalized by the length of the vector $\left(\bar{S_{1}},\bar{S_{2}},\bar{S_{3}}\right)$ so that

Si¯^=Si¯S1¯2+S2¯2+S3¯2. (5)

Accordingly, the reciprocal of the resulting first component provided the degree of polarization (DOP)^37^ as

DOP=S1¯2+S2¯2+S3¯2S0¯=1S0¯^. (6)

By calculating the DOP in this order, i.e., first creating an incoherent ensemble by spatial averaging from which the depolarization effect of the sample can be derived,^25^ the result is physically corresponding to the measurement of partially or unpolarized light for incoherent imaging techniques. Nevertheless, the result is similar to what has been described as DOP uniformity.^39^

Now, each three-component set of Stokes parameters S→=(S1¯^,S2¯^,S3¯^) has a length of one, i.e., $\|\overset{\to }{S}\|=1$, due to the manner in which the normalization is performed. To visualize the local polarization change, the included angle $\alpha =∢(\overset{\to }{S}(z),\overset{\to }{S}(z+\Delta z))$ between two subsequent Stokes parameter sets was calculated, which represents the rotation of the Poincaré sphere to directly transform one state into another.^37^ Due to the normalization, the scalar product

$$\langle \overset{\to }{S}(z),\overset{\to }{S}(z+\Delta z)\rangle =\|\overset{\to }{S}(z)\|\cdot \|\overset{\to }{S}(z+\Delta z)\|\cdot \cos\;\alpha$$ (7)

can be used to directly determine α, which is otherwise described as the phase retardation in case of orthogonality between the incident polarization and the optic axis of the birefringent media on the Poincaré sphere. Accordingly related to the axial step size Δz of 7.8  μm and scaled by the multiplication with the central wavelength $\lambda_{0}=1300\;\mathrm{nm}$ over 2π, and over 2 to account for the round trip,

$$\Delta n_{\mathrm{app}}(z)=\arccos\langle \overset{\to }{S}(z),\overset{\to }{S}(z+\Delta z)\rangle \cdot \frac{\lambda_{0}}{4\pi \cdot \Delta z}$$ (8)

provides the apparent local retardation. This method is able to provide the correct local retardation, e.g., when circularly polarized light is incident on the sample, which is $\Delta n=n_{\mathrm{eo}}-n_{o}$ the difference of the ordinary $n_{o}$ and the extraordinary refractive index $n_{\mathrm{eo}}$.^37^ However, it tends to underestimate the actual amount of birefringence for converging incident state and optic axis such that $\Delta n_{\mathrm{app}}\leq \Delta n$. Compared with other approaches based on a single polarization input state, this evaluation is rather a pseudolocal approach than an accurate determination. While including a correction for this dependency using symmetry constraints of the round-trip signal^40^^,^^41^ or a polarization state tracing^42^^,^^43^ that takes into account multiple axially subsequent states, the method, proposed here, requires less information to derive an estimate.

### 2.4 Visualization

**Fig. 3:**
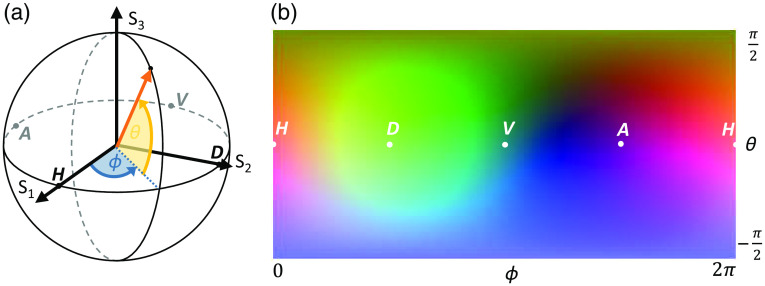
RGB color map to visualize Stokes polarization states. Polarization states as represented on the surface of the (a) Poincaré sphere with RGB values corresponding to three Stokes parameters were mapped to the (b) projected surface of the sphere based on the azimuth ϕ and elevation angle θ. H, D, V, and A are indicating linear polarization states with horizontal, diagonal, vertical, and antidiagonal orientation, respectively.

As the polarization state of light represented by the Stokes parameters in $\vec{S}$ forms a 3D space, which is the surface of the Poincaré sphere, we used the RGB color space to visualize the measured polarization states. Therefore, the normalized Stokes components $\left(\hat{S_{1}},\hat{S_{2}},\hat{S_{3}}\right)$ were scaled from $\hat{S_{i}}=[-1,1]$ to the RGB color space $\hat{S_{c}}=(\frac{\hat{S_{i}}}{2}+0.5)\cdot 255$, where i=(1,2,3)→c=(R,G,B), similar to the mapping in Ref. 44. Accordingly, each of the three Stokes parameters corresponds to one color channel that is the red channel to $\hat{S_{1}}$, the green one to $\hat{S_{2}}$, and the blue one to $\hat{S_{3}}$. To visualize the mapping of RGB colors and polarization states in a 2D representation (Fig. 3), the Stokes parameters on the Poincaré sphere (left) were transformed to a spherical coordinate system and plotted depending on the azimuth ϕ and elevation angle θ.

**Fig. 4:**
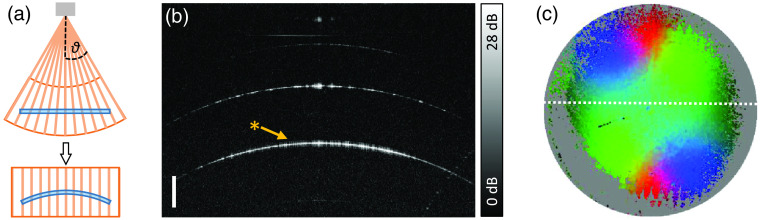
(a) Imaging through the GRIN endoscope induces a fan-shaped distortion that causes the plane surface of a microscope slide to appear curved. (b) The distribution of the Stokes parameters calculated from the light, which was reflected at the bottom side of a microscope slide (*), is visualized using (c) the RGB color map. The scale bar corresponds to 1 mm in air.

As sketched in the simulated beam path of Fig. 1, the GRIN objective lens at the distal end of the endoscope causes a fan-shaped scan pattern and thus distortion of the acquired data [Fig. 4(a)]. To visualize the effect of the GRIN optics used in the current setup, a reference measurement of a microscope slide (thickness d=1  mm) was done. In Fig. 4, a cross-sectional image (b) shows a central scan of the volume as marked by the dashed white line in the *en face* image (c). The curvature of the measured signal illustrates the distortion caused by the fan-shaped scanning of the plane microscope slide (a). Although this distortion can be corrected by an appropriate geometrical transformation as previously described^2^ and applied for visualizing the entire volume in Fig. 2, the distorted representation of the raw images is preferred in the following. Because the presented results depend on the incident angle on the surface, representing the beam direction always vertically oriented in the raw images is advantageous in this case to visualize the local incidence. In addition, spatial averaging was applied in the distorted voxel scale rather than in the corrected geometric relations. To consider the aspect ratio at the working distance, however, distorted images are displayed assuming a lateral size of 10 mm, which is most appropriate in the axial center of the images.

Furthermore, the polarization states were evaluated [Fig. 4(c)] that are expected to correspond to the states incident on the sample, which should be similar to signals that were back-reflected from the microscope slide [* in Fig. 4(b)]. Due to the vanishing signal toward the border of the FOV, the center was mainly evaluated, and the gray area indicates the entire FOV. While the back reflection of the glass surface as well as the slide itself is polarization maintaining, a symmetric variation of the measured polarization state over the FOV with central inversion was observed, which is a result of the birefringent GRIN optics. Thus, any evaluation of the directly measured polarization states needs to consider a spatial variation regardless of the sample-induced birefringence. Otherwise, a basic evaluation without further considerations can be misleading as it shows the cumulative effect including the GRIN optics.

**Fig. 5:**
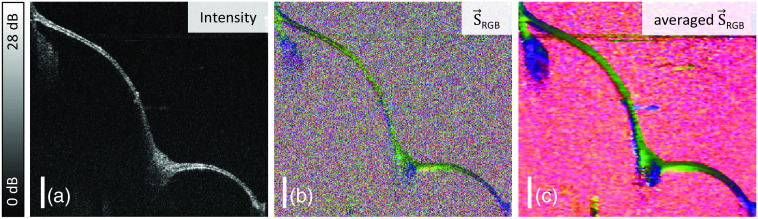
Cross sections of the TM from the measurement and position shown in Fig. 2 are presented based on the (a) intensity signal as well as the RGB-color encoded Stokes parameters (b) without and (c) with averaging. Scale bars correspond to 1 mm in air.

**Fig. 6:**
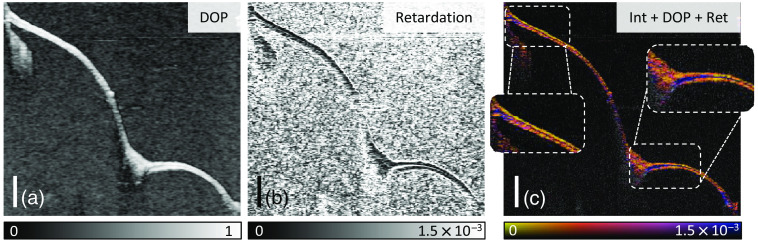
Cross sections of the TM from the measurement and position shown in Fig. 2 are presented based on the (a) DOP and (b) apparent local retardation signal as well as the merged representation of (c) intensity, DOP, and color-encoded retardation signal. Scale bars correspond to 1 mm in air.

Similarly, the Stokes parameters were used for visualizing the *in vivo* measurement of a right TM (Fig. 5). (a) Besides the intensity signal of the same cross section as presented in Fig. 2, Fig. 5 compares the RGB color-encoded Stokes parameters (b) without and (c) with spatial averaging in accordance with Eq. (4). To better assess the local effects, the DOP [Eq. (6)] and the differential Stokes signal [Eq. (8)], which corresponds to the local retardation, were calculated. Figure 6 shows the resulting (a) DOP and (b) retardation signal with a clear differentiation of the depolarized background in comparison to the mainly high DOP of the TM signal. As only regions of sufficient intensity signal, i.e., considerably above the noise floor, and for a DOP>0.5 are meaningful in terms of the local retardation contrast, all modalities were merged in one representation as shown in Fig. 6(c). Here the intensity-overlaid retardation and the DOP are combined so only regions with DOP>0.5 appear as a color-encoded representation of the retardation signal. In detail, the retardation signal was colored using a map that is also perceptible in case of a red-green deficiency.^45^ Then a mask generated from an exponential slope for fading out the color values for pixels with a DOP < 0.5 was applied so that the color-encoded retardation signal is only displayed for sufficient DOP values. Eventually, the result was combined with the intensity image as brightness.

## 3 Results

Figure 6 presents the results of the same *in vivo* examination of a right human TM that is also visualized in Fig. 2 including the volumetric dataset and the camera image. The cross-sectional image [Fig. 6(c)] illustrates the additional contrast derived from the polarimetric evaluation of the OCT data compared with the intensity-based image in Fig. 5(a) with insets on the most prominent regions. Opposing this differential representation to the cumulative Stokes parameters in Fig. 5, the interpretation of the polarization change within the tissue is more clearly facilitated in the calculated local retardation, although in the averaged Stokes parameters [Fig. 5(c)] a color shift in axial direction, which corresponds to a rotation of the Poincaré sphere, allows identifying different regions. While the cumulative representation does show all polarization changes affecting the light on its path through the optical setup and the sample, the differential evaluation indicates the regions that induce polarization changes due to birefringence. In general, the interpretation of the color gradients of the Stokes images might be misleading especially because the GRIN optics causes varying surface polarization states and thus colors, which is otherwise compensated by the differential contrast. A distinct retardation signal is visible in Fig. 6(c) indicated by the blue color ($\Delta n_{\mathrm{app}}=1.5\times 10^{-3}$) most clearly in the highlighted regions, mainly close to the umbo and in the region near the annulus, where also an increased thickness of the TM can be typically observed.^13^ In both regions, this reveals a birefringent tissue layer within nonbirefringent tissue, which corresponds to the studies conducted with electron microscopy and thin sections. ^9^^–^^11^ Concurrently, these regions of high retardation show a lower DOP, which is to some extent corresponding to the determined retardation. As an interferometric technique does only account for fully polarized light, the DOP in PS-OCT is generally based on Stokes averaging of adjacent speckles, thus creating an incoherent ensemble. Local retardation, however, implies axially changing Stokes parameters and calculating the mean thereof accordingly a DOP<1. The additional lateral averaging otherwise has an effect similar to depolarization in case of steep surfaces, which is apparent in the posterior inferior region from the umbo outward. While this is also reasonably true in the annulus region [left inset in Fig. 6(c)] with significant retardation, a relatively flat surface with respect to the incident beam was observed near the umbo (right inset). In general, more complex birefringent structures, e.g., an interwoven pattern of fibers with different optic axes that cannot be resolved might be the origin of depolarization. There is no continuous retardation signal visible across the whole TM. In some thinner regions, very narrow retardation bands occur in the order of the axial resolution limit. Due to this limitation, the differential processing of the spatial average is fundamentally not capable of analyzing all TM regions when an average minimum thickness of 112  μm of the entire membrane *in vivo* is assumed,^14^ whereas the resolving power is limited to ∼27  μm.

Besides the described depolarizing effects, in the area of steep slopes and thus unfavorable beam angles of the light onto the TM’s surface, the retardation signal vanished. While this signal decrease comes in concurrence with the lower back-scattering intensity signal due to the incident angle, also the spatial alignment of beam incidence and 3D optic axis orientation are detrimental. As the polarization-sensitive measurement is always a projection of the 3D vectorial birefringence onto the plane perpendicular to the incident beam, the effective retardation approaches zero when optic axis and incidence converge. In consequence, the measurements of steep regions are especially difficult to evaluate.

**Fig. 7:**
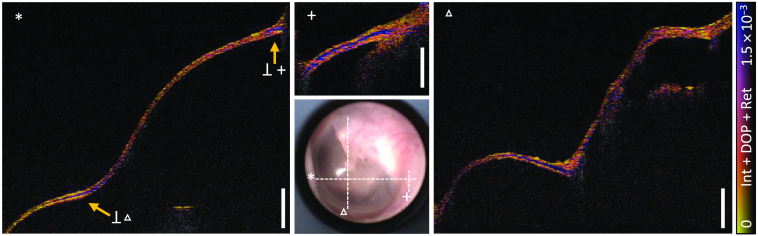
Cross-sectional images of the left ear *in vivo* at the positions marked in the camera image show the merged representation of intensity, DOP, and color-encoded retardation signal. The cross section marked with (*) corresponds to the fast scanning axis, the cross section labeled (+) and (Δ) to the slow scanning axis. Points of orthogonal intersection of fast and slow axis scans are indicated in (*) by an arrow. Scale bars correspond to 1 mm in air.

Further evaluation showed the presence of high retardation signals in the left ear volume consistently as demonstrated in Fig. 7. The positions and ranges of the cross-sectional images are marked in the camera image. The slice corresponding to the fast-scanning direction (*) exhibits a clear retardation signal despite that the membrane is thin in the inferior region. The central frame acquired in the slow-scanning direction (+) originates from an annulus region crossing the left slice (*) perpendicularly at the indicated position. Accordingly, the finding of significant retardation in the annulus region is consistent in the surrounding area. In the right cross section (Δ), again in slow-scanning direction, the contrast is visible in the region close to the umbo, thus in agreement with the signal found in the crossing plane. No clearly visible layer was detected nearby the connection to the malleus, which showed otherwise a reduced intensity. A more complex interwoven fiber structure is probably underlying, which cannot be distinguished, whereas a distinct connection between the surrounding fibers and the malleus is to be expected due to the acoustomechanical importance. At the right border of the image (Δ), the scan intersected the region of the pars flaccida, which is a part of less tension and thus presumably not as enforced with collagen fibers as the pars tensa, the main part of the TM. Accordingly, there was also no significant retardation signal detected. Directly beneath this TM part, the signal was identified as a part of the malleus.

**Fig. 8:**
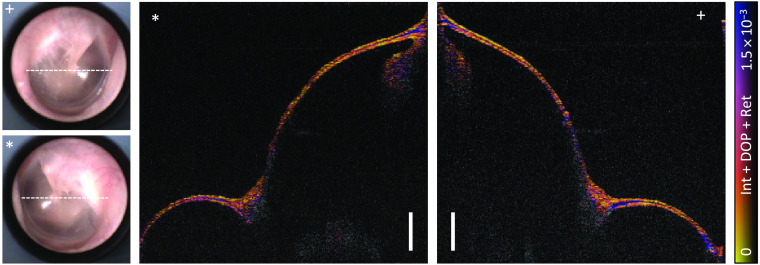
Comparison of a left (*) and a right (+) TM based on the color-encoded retardation representation. Markings in the camera images (left) indicate the shown cross sections. Scale bars correspond to 1 mm in air.

While Figs. 6 and 7 referred to measurements of the right and left ear of the same volunteer, the volumes were furthermore compared directly. Besides minor differences, the retardation signals were detectable for both ears symmetrically as displayed in Fig. 8. The strongest signal appeared in the thickest regions at the umbo and the annulus for both ears although the movement of the endoscopic probe in between the measurements had caused a change of the SMF birefringence and thus different incident polarization states for each of the measurements. In addition, the curvature and thickness were identical to a great extent while also in the camera image characteristic features, e.g., the light reflex, appeared symmetrically.

## 4 Discussion

Compared with the results of GRIN-based *in vivo* endoscopic OCT^2^ presented before, the image quality and thus the feasibility of further evaluations increased considerably. For this purpose, using a different light source was decisive, which did not only speed up the measurements due to a higher sweep rate but also eliminated artifacts originating from the GRIN endoscope interfaces and the coherence revival effect of the previous laser. Furthermore, an increased depth range allowed measuring the whole TM reliably and even structures behind it regardless of the individual geometry as it was considered to be of relevance during concurrent experiments.^14^^,^^33^

Furthermore, the modification of the revised system to be a polarization-sensitive instrument was also advantageous for the intensity-based imaging by itself. Evaluating the total intensity as sum of the intensities detected from both orthogonal polarization channels while controlling a diagonally linear polarization in the reference arm prevented polarization-dependent extinction of the interferometric signal, which can otherwise result from the mismatch between the polarization states of the interferometer arms.

The investigation shows that the usage of endoscopic PS-OCT enables identifying birefringent tissue of the human TM *in vivo*. Significant retardation signals in the annulus region and close to the umbo are in good agreement with the available anatomical studies about the layered structure of the TM and in particular collagen fibers in the lamina propria,^9^ whose birefringence and orientation are expected to induce polarization changes. Thus the detected retardation refers indirectly back to the collagen fiber content of the TM. The measured order of retardation of about $\Delta n_{\mathrm{app}}=1.5\times 10^{-3}$ is reasonable in comparison with other birefringent tissues examined with PS-OCT, e.g., oral mucosa of the inner lip,^26^ breast tumor samples,^30^ or coronary artery wall.^28^

Nevertheless, based on previous light and electron microscopic surveys,^9^ as well as *ex vivo* PS-OCT measurements,^31^^,^^32^ birefringent tissue is expected to appear across the entire membrane, which contrarily did not arise in the current measurements and evaluations, thus raising the question about the measurement limitations.

In general, the examination of the TM is relatively constrained due to the shape and limited flexibility of the ear canal. The conical form of the TM and its tilted position at the end of the ear canal imply that a significant part of surface area is examined under a steep angle, even though fan-shaped scanning was used. While the marginal areas of the TM and the anterior region around the umbo provide the best back-scattering signal due to an almost perpendicular illumination, the incoming beam approaches a nearly parallel incidence with respect to the TM’s surface in the posterior half. For such large angles, even larger than 70 deg, the polarization of the light penetrating the tissue is changed as described by the transmission coefficients of the Fresnel equations, which is effectively diattenuation. Although this contribution was not considered in the applied processing, using multiple input states would facilitate to determine the diattenuating behavior.^46^

In addition, the thickness of the lamina propria tends to correlate with the overall membrane thickness, as the regions of high retardation correspond to the thickest regions of the TM as stated by Van der Jeught et al.^13^ While preliminary *ex vivo* PS-OCT measurements seem to support the presumed increased thickness of the collagen layer,^32^ further interpretations of the collagen layer thickness distribution are limited due to a lack of fundamental knowledge and missing investigations, especially *in vivo*. Accordingly, there is a need for more comprehensive *ex vivo* measurements with both higher resolution and appropriate positioning of the temporal bone specimen to compensate for the regularly tilted TM in the ear canal. Although increasing the lateral resolution is essentially limited by the geometry of the ear canal and a necessarily safe working distance, merely enhancing the axial resolving power should be highly beneficial *in vivo*. As the reflexes occurring at the surfaces, i.e., the border between air and tissue and vice versa, generate dominant signals that overlap with the less intensely scattering inner layer, axially separating those contributions promises considerable improvements.

Additionally, the current axial resolution limit of the system and processing of ∼27  μm might be not sufficient for the detection of thin collagen layers as they seem to appear in the TM. Therefore, a resolution-preserving despeckling of the Stokes parameters can provide further improvements.

Similarly, a more sophisticated polarization processing can be more exhaustive than the simplified approach shown here. While solutions such as the mirror-state approach,^40^^,^^41^ which includes spectral binning^28^ or polarization state tracing,^42^^,^^43^ can improve the evaluation of single-input state measurements, using multiple polarization input states, e.g., by a modulation scheme^29^ or a polarization-delay unit,^46^ might be advantageous. However, the latter concept would require a manifold-extended imaging range or suffer from an interleaving of the TM signal of one polarization state with middle ear structures of the other polarization. Otherwise using polarization-maintaining fibers and a circular illumination, which ensures the optimal contrast at least in the first birefringent layer, is not convenient in this setup due to the detrimental GRIN lens birefringence causing different polarization states across the FOV.

Endoscopic PS-OCT has the potential to serve as a diagnostic tool in middle ear cases that benefit from the ability to discriminate tissues or structures more specifically than it is feasible through the intensity signal. Even without pathological alterations, e.g., for intraoperative imaging and surgery guidance when placing a prosthesis optimally, a better recognition of structures is beneficial. Among the assessment of pathological changes, the diagnosis of tympanosclerosis is a promising application as it causes the calcification of connective tissue, which might be detected by characteristic polarization signals, i.e., reduced birefringence or depolarization. Presumably, the demarcation of, for example, sclerotic tissue from the ossicular bones or for better evaluating the pathological condition based on the degradation of the fiber structure can be visualized. Similarly, those advantages apply for the evaluation of tympanoplasty outcome.^33^ In chronic cases of otitis media, PS-OCT could support the differentiation of the inflamed membrane’s tissue from the incorporated biofilm and thus facilitate clinical staging and therapy assessment. Finally, TM reconstructions could profit from the possibility to determine the collagen content and its thickness distribution for creating improved artificial replica. The gained knowledge could be used in the attempts to mimic the TM lifelike by bioprinting^20^^,^^21^ or later on even for patient-individual adaptations. Yet, the actual clinical application of the endoscopic PS-OCT still needs to be validated by conducting further *in vivo* investigations of both healthy and pathologically altered TMs.

Overall, this sum of application opportunities, even though not crucial, combined with the minor complexity of redesigning the OCT system for being polarization sensitive as shown here and accordingly the manageable effort for clinical translation, should be considered when thinking about introducing OCT in otolaryngology.

## 5 Conclusion

Current diagnostic and imaging methods lack the ability to assess the microstructure of the human TM *in vivo*. While its layered structure and approximate distribution of connective tissue are described in the available literature, which is mainly based on *ex vivo* investigations, both clinical and preclinical applications would benefit from an imaging method capable of directly examining the TM with tissue-specific contrast *in vivo*.

In this study, an endoscopic PS-OCT was developed and its capability of differentiating the birefringent from nonbirefringent tissue in the human TM *in vivo* is investigated. While regions of significant retardation signals were in agreement with the anticipated occurrence of a thicker fibrous layer, i.e., near the annulus ring and in the surroundings of the umbo, it was not possible to track collagen-related signals across the entire TM. In general, the geometry of the TM within the ear canal poses a challenge by almost parallel surfaces with respect to the incident beam in certain regions. Therefore, future improvements are primarily expected in the determination of the local retardation contrast and the birefringence evaluation. As the described system extension toward PS-OCT was comparably effortless, enhancing a proposed OCT system with polarization-diverse detection might be considered not only if the additional information is specifically required but also for fundamental insights. In the case of middle ear imaging, further *in vivo* studies are required to gain an extended knowledge about the polarization dependency of pathological changes as well as healthy tissue, which will eventually lead to a comprehensive review of the clinical potential.

## Data, Materials, and Code Availability

All relevant data, materials, and software code used in this research are available upon request from the corresponding author.
